# Validation of an apnea and hypopnea detection algorithm implemented in implantable cardioverter defibrillators. The AIRLESS study

**DOI:** 10.1038/s41598-019-45255-3

**Published:** 2019-07-03

**Authors:** Pascal Defaye, Monique Mendelson, Renaud Tamisier, Peggy Jacon, Sandrine Venier, Nathalie Arnol, Jean-Louis Pépin

**Affiliations:** 1 Cardiology Department- University Hospital of Grenoble Alpes and INSERM U 1039, Grenoble, France; 2 HP2 laboratory, INSERM U1042, University Grenoble Alpes, Grenoble, France; 3 EFCR Laboratory, Grenoble Alpes University Hospital, Grenoble, France

**Keywords:** Cardiac device therapy, Interventional cardiology, Ventricular fibrillation

## Abstract

Diagnosis of sleep apnea (SA) using simple tools has the potential to improve the efficacy of cardiac implants in the prevention of cardiac arrhythmias. The aim of the present study was to validate a transthoracic impedance sensor for SA diagnosis in patients with cardiac implants. We compared the apnea-hypopnea index (AHI) obtained from polysomnography (AHI_PSG_) with the AHI obtained from autoscoring algorithms of the ApneaScan implantable impedance respiration sensor (AHI_AS_) three months after implantation of cardioverter-defibrillator (ICD) or cardiac resynchronization therapy-defibrillator (CRT-D) devices. Twenty-five patients with indications for implantation of ICD or CRT-D (INCEPTA; Boston Scientific) (24 men, 59.9 ± 14.4 years; LVEF 30.3 ± 6.4%; body mass index 25.9 ± 4.2 kg/m²) were included. Mean AHI-_PSG_ was 21.9 ± 19.1 events/hr. A significant correlation was found between AHI_PSG_ and AHI_AS_ especially for the most severe SA (Spearman correlation: 0.71, p < 0.001). Intraclass Correlation Coefficient (was in the expected range: 0.67, 95% CI: 0.39–0.84. The mean bias was 5.4 events per hour (mean AHI: 23.3 ± 14.6 versus 29.7 ± 13.7 for AHI-_PSG_ and AHI-_AS,_ respectively). An optimal cutoff value for the AHI_AS_ at 30 events/h was obtained from the Receiver Operator Characteristic (ROC) curve analysis, which yielded a sensitivity of 100%, a specificity of 80%, PPV = 67%, NPV = 100%. Using an advanced algorithm for autoscoring of transthoracic impedance included in ICDs is reliable to identify SA and has the potential to improve the management of patients with cardiac implants.

## Introduction

Sleep-disordered breathing (SDB), most commonly presenting as either obstructive sleep apnea (OSA) or central sleep apnea (CSA) with Cheyne–Stokes respiration, has been recognized as a risk factor for cardiovascular diseases such as hypertension, coronary heart disease and heart failure (HF)^1^. The prevalence of SDB in patients with cardiovascular disease is high and might contribute to modulating evolution and prognosis^1^. The complexity of a standard nocturnal polysomnography (PSG) performed in the laboratory for sleep apnea identification imposes a large patient burden and a relevant economic cost to the healthcare system and may limit the access to diagnosis^1^.

New techniques for diagnosing sleep apnea (SA) have been proposed for specific at risk patient populations. Pacemaker transthoracic impedance sensors were used to facilitate SA diagnosis in chronically implanted patients^2–4^. However, one of the challenges was the low reliability of the systems to identify sleep duration. The identification of sleep disorders can now be automatically achieved by algorithms that incorporate physical activity monitoring, thus allowing to separate daily activity and sleep periods. This might improve the sensitivity of SA diagnosis by cardiac implants. Additionally, an apnea-hypopnea detection algorithm has recently been implemented in implantable defibrillators (ICD and CRT-D, model INCEPTA; Boston Scientific) and can be used in patients at high risk of sudden death.

The objective of the current study was to compare the apnea-hypopnea index (AHI) obtained from conventional in-lab PSG (AHI-_PSG_) with the autoscoring algorithm (the ApneaScan) implemented in ICDs through the impedance respiration sensor three months after ICD or CRT-D implantation.

## Methods

The present study was a prospective, multi center (Grenoble, Nancy, Montpellier), phase-IV with blinded analysis and central reading of polysomnography trial. The target population for the AIRLESS study was the general patient population indicated for an ICD. Heart rate and left ventricular ejection fraction (LVEF) were monitored at baseline, 6 weeks, and 12 weeks after ICD or CRT-D implantation. The study was conducted in accordance with applicable good clinical practice requirements in Europe, French Law, ICH E6 recommendations, and Ethical Principles of the Helsinki Declaration. It was approved by an independent Ethics Committee (Comité de Protection des Personnes, Grenoble, France, IRB0005578) and registered on the ClinicalTrials.gov site (NCT02979184). Written informed consent was obtained from all patients.

### Inclusion and exclusion criteria

Patients with a standard CT-D or ICD^5^ indication were eligible for enrolment in the study. They were implanted with devices implemented with the ApneaScan algorithm. All patients were able to manage the Remote Monitoring System procedure (LATITUDE Patient Management System) for automatic data transmission.

We excluded patients with obstructive lung disease as defined by a FEV1/FVC less than 70%, body mass index greater than 35 kg/m^2^, patients scheduled for cardiac surgery or who had a strong likelihood of cardiac surgery 4 months after enrolment.

### Polysomnography

OSA diagnosis was obtained by full polysomnography. Sleep was scored manually according to standard criteria^6^. Polysomnography used continuous acquisition of the following recordings: electroculogram (EOG; 3 channels), electroencephalogram (EEG; 3 channels), electromyogram (EMG; 1 channel) and electrocardiogram (ECG; 1 channel). Airflow was measured using nasal pressure associated with the sum of oral and nasal thermistor signals. Respiratory effort was monitored with abdominal and thoracic bands. An apnea was defined as a complete cessation of airflow ≥ 10 s and a hypopnea as a reduction ≥ 50% in the nasal pressure signal or a decrease between 30% and 50% associated with either oxygen desaturation ≥ 4% or EEG arousal. Apneas were classified as obstructive, central, or mixed according to the presence or the absence of respiratory effort. The criterion for sleep apnea in this study was an apnea-hypopnea index (AHI) ≥ 15 events per hour of sleep, as described previously^7^. PSG was performed in hospital at baseline before implantation and 3 months after device implantation.

### Sleep Apnea algorithm and data transmission system

**Figure 1:**
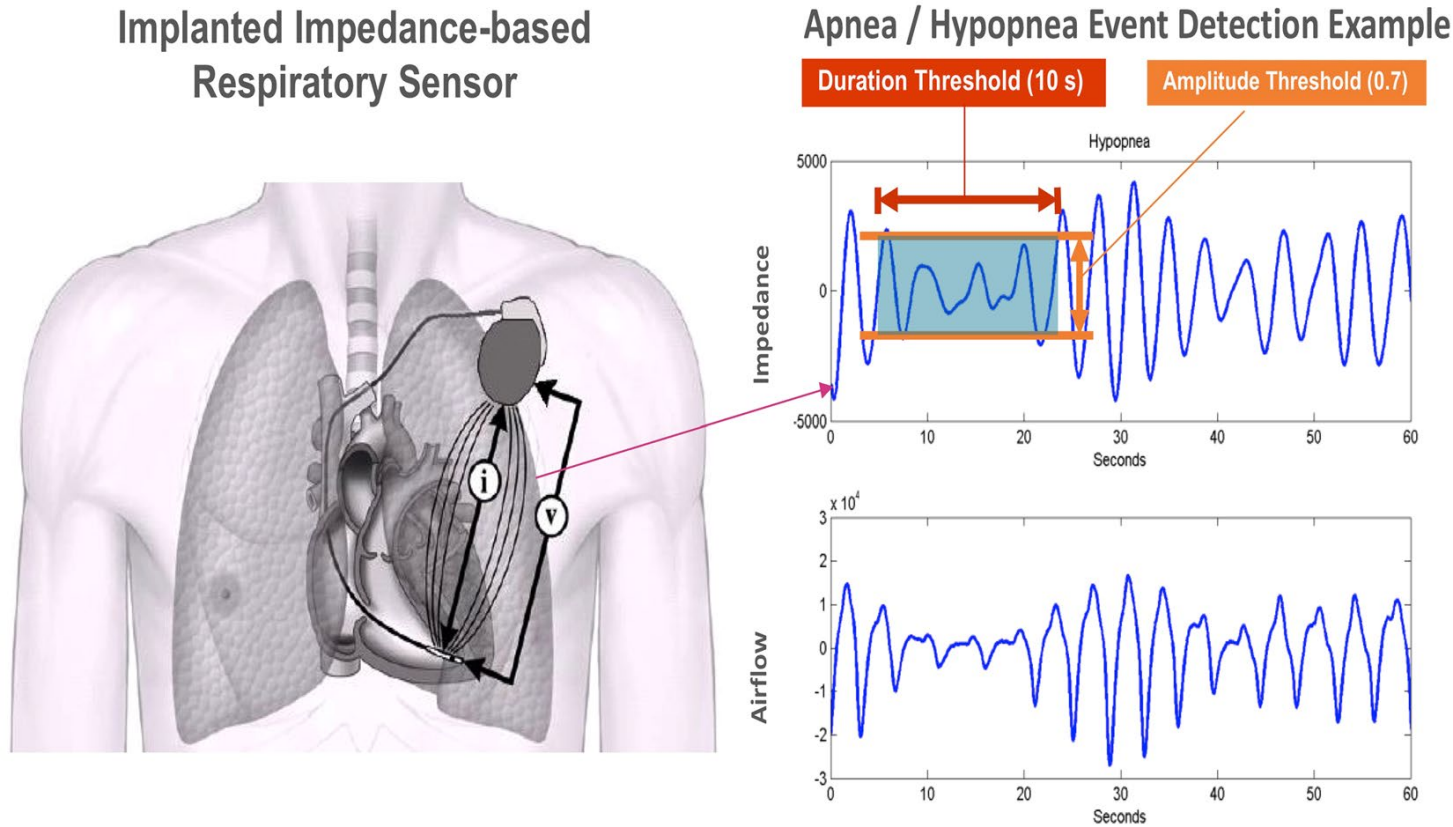
The Apnea Scan algorithm implemented in the ICD monitors within the patient’s core sleep hours, the SDB, expressed as Respiratory Disturbance Index (RDI). The device reports only the severe sleep apnea (RDI ≥ 32) as a trend. Material provided courtesy of Boston Scientific. Copyright 2019 © Boston Scientific Corporation or its affiliates. All rights reserved.

The ApneaScan system is an impedance-based respiratory sensor implemented in the INCEPTA devices (ICD and CRT-D). It monitors within the patient’s core sleep hours, the SDB, which is expressed as Respiratory Disturbance Index (RDI) (Fig. 1).

The LATITUDE Patient Management System is used to remotely connect clinicians and patients to support cardiac care, through a Communicator. The Communicator uses a radio frequency communication system to send and receive signals from the implanted device and to communicate with optional weight scale and blood pressure monitor. The information from the devices is sent to a secure website: LATITUDE. It can only be viewed by the healthcare support team.

The system is designed to inform the physician within 24 hours if new pulse generator alert conditions are detected by the Communicator.

### Statistical analysis

Normality of the data was tested with Skewness and Kurtosis tests and equality of variance was assessed by Levene’s test. Data were expressed as median and inter-quartile range [25%; 75%] when appropriate. Comparisons between AHI-_PSG_ and AHI-_AS_ were expressed as proposed by Bland and Altman^8^. Correlation was assessed by Pearson’s or Spearman’s coefficients depending upon the normality of data distribution. A receiver operating characteristic (ROC) curve analysis was conducted to assess the performance of the AHI-AS as a predictor for SA (determined by the sleep study on the basis of an AHI cutoff of 30 episodes/h). For all tests, a level of significance of p < 0.05 was considered. Statistical analysis was performed by a biostatistician. with NCSS 97 software (Kaysville, Utah, USA), SAS 9.1.3 software (SAS Institute, Cary, NC, USA) and SPSS Statistics 17 (Chicago, USA).

## Results

### Study population

**Figure 2:**
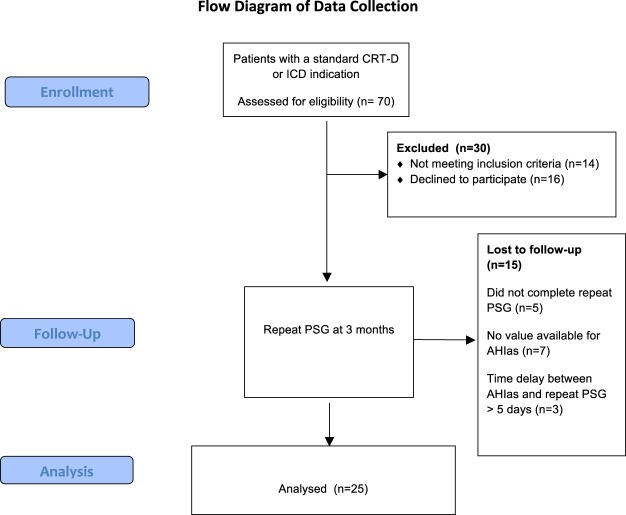
Flow diagram of data collection.

The flow of participants is presented in Fig. 2. Twenty-five patients (24 men, mean age 59.9 ± 14.4 years; LVEF 30.3 ± 6.4%; body mass index 25.9 ± 4.2 kg/m^2^) were enrolled in the study. Sixteen patients (70%) were implanted for primary prevention of sudden cardiac death. Nine patients (36%) were implanted with a CRT-D. Twelve patients out of 25 (48%) were affected by coronary artery disease. Twelve patients (48%) had clinical history of atrial fibrillation. Six patients (24%) were in NYHA class III-IV (Table 1).

**Table 1:** Demographics and baseline clinical parameters.

Parameter	Mean (SD)
Male gender, n(%)	24 (96)
Age (years)	59.9 (14.4)
BMI (kg/m^2^)	25.9 (4.2)
Apnea hypopnea index (events/hr)	21.9 (19.1)
LVEF (%)	30.3 (6.4)
Coronary artery disease n(%)	25(48)
Atrial fibrillation n(%)	12 (48)
NYHA class III-IV n(%)	6 (24)

### Sleep study

The mean AHI on polysomnographic recordings was 21.9 ± 19.1 events/hr. Seven patients (28%) had CSA and six had OSA (24%). Severe SA was diagnosed by AHI-_PSG_ in 8/25 (32%) of the studied patient population.

### Primary outcome

**Figure 3:**
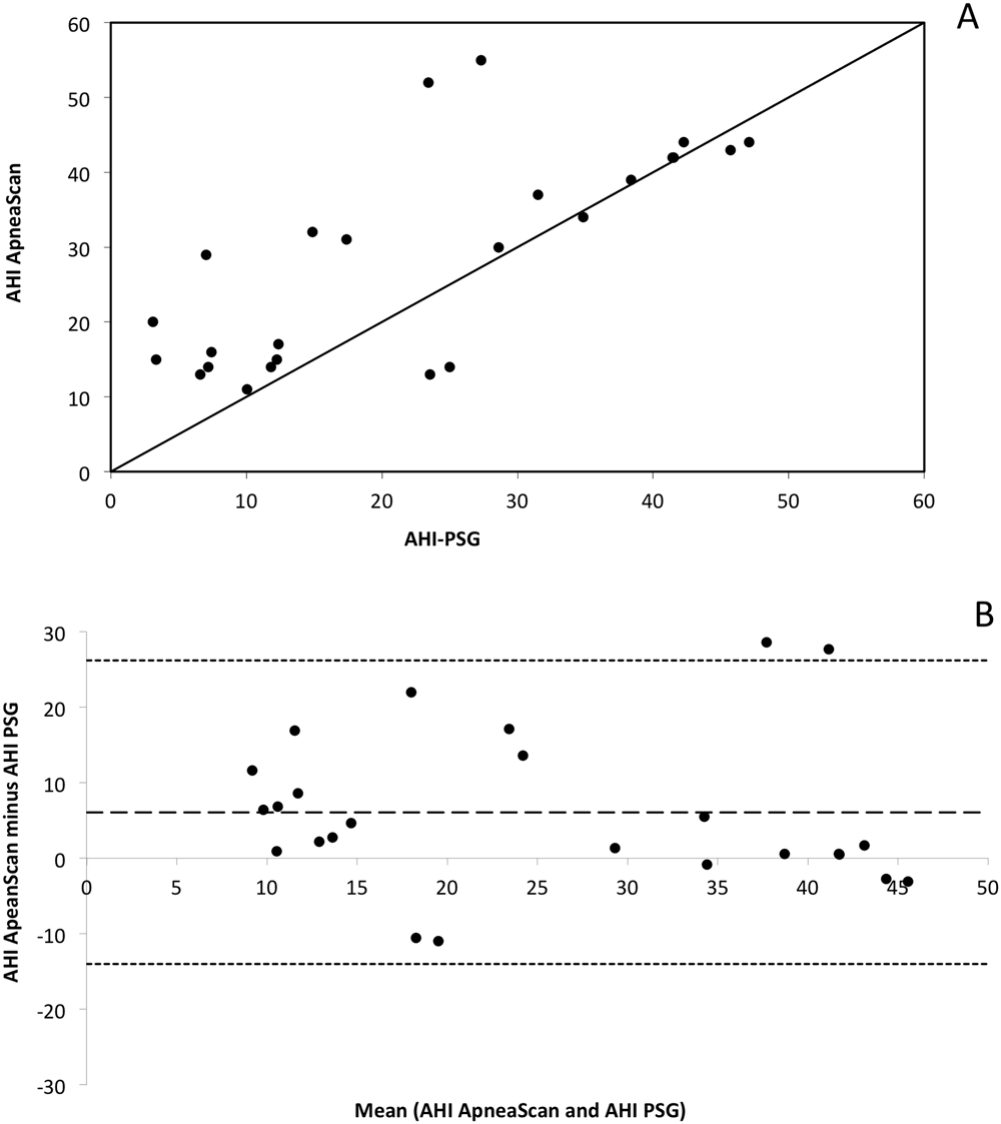
A statistically significant correlation was found between AHI-PSG and AHI-AS for the most severe SA: Spearman Correlation Coefficient = 0.71, p < 0.001. The Intraclass Correlation Coefficient (ICC) was in the expected range 0.67, 95% CI: 0.39–0.84. The mean bias on the Bland-Altman analysis was 5.4 events per hour (mean AHI: 23.3 ± 14.6 versus 29.7 ± 13.7 for AHI-_PSG_ and AHI-_AS,_ respectively).

A statistically significant correlation was found between AHI-_PSG_ and AHI-_AS_ for the most severe SA: Spearman Correlation Coefficient = 0.71, p < 0.001) (Fig. 3A,B).

**Figure 4:**
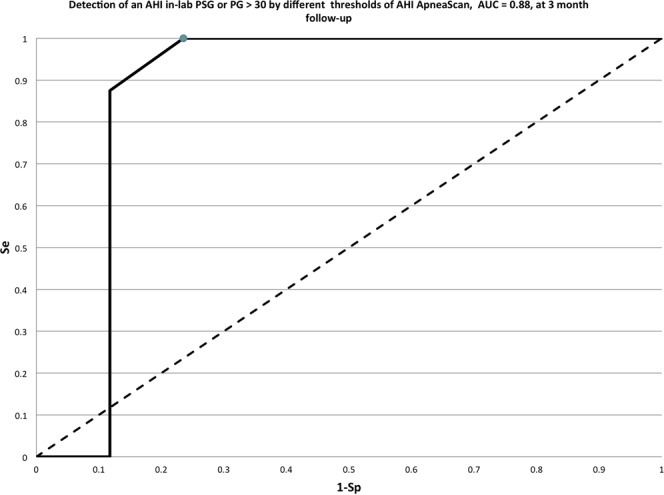
R.O.C. curve corresponding to the data on Table 2. With a threshold of 30 for AHI-AS, the corresponding Sensitivity is 100%, Specificity 76.47%, PPV 66.67%, NPV 100%.

The Intraclass Correlation Coefficient (ICC) was in the expected range: 0.67, CI: 95%, range 0.39–0.84. The mean bias on the Bland-Altman analysis was at 5.4 events per hour (mean AHI: 23.3 ± 14.6 versus 29.7 ± 13.7 for AHI_PSG_ and AHI_AS,_ respectively). An optimal cutoff value for the AHI_AS_ at 30 events/h was obtained from the Receiver Operator Characteristic (ROC) curve analysis, which yielded a sensitivity of 100%, a specificity of 80%, PPV = 67%, NPV = 100% (Fig. 4, Table 2).

**Table 2:** Detection of AHI psg greater than 30 versus different thresholds of AHIas, at 3 month follow-up.

AHI as, thresholds	TP: AHI psg > 30 and AHI as >threshold	FP: AHI psg ≤ 30 and AHI as >threshold	FN: AHI psg ≥ 30 and AHI as ≤thrshold	TN: AHI psg ≤ 30 and AHI as ≤threshold	Se	Sp	PPV	NPV
10	8	17	0	0	100,0	0,0	32,0	/
15	8	9	0	8	100,0	47,1	47,1	100,0
20	8	6	0	11	100,0	64,7	57,1	100,0
25	8	6	0	11	100,0	64,7	57,1	100,0
**30**	**8**	**4**	**0**	**13**	**100**,**0**	**76**,**5**	**66**,**7**	**100**,**0**
35	7	2	1	15	87,5	88,2	77,8	93,8
40	5	2	3	15	62,5	88,2	71,4	83,3
45	0	2	8	15	0,0	88,2	0,0	65,2
50	0	2	8	15	0,0	88,2	0,0	65,2
55	0	0	8	17	0,0	100,0	/	68,0

Abbreviations: TP: true positive; FP: false positive; FN: false negative; TN: true negative; Se: sensitivity; Sp: specificity; PPV: positive predictive value; NPV: negative predictive value.

## Discussion

The present study demonstrated that an automatic algorithm implemented in ICDs can reliably detect sleep apnea. The major finding of the study is the strong correlation we showed between the RDI from ApneaScan algorithm and polysomnography, the gold standard diagnostic tool for sleep apnea. In addition, the sensitivity, specificity, PPV, and NPV, are clinically relevant for the use of this system in standard clinical practice. The reliability of our data is reinforced by a core lab central scoring and a clear differentiation between central and obstructive events.

Some recent publications have added significant information about the detection of SA in patients with cardiac implants^2,9–13^. D’Onofrio *et al*.^9^ recently evaluated the ApneaScan algorithm in 265 patients with implantable ICDs and assessed the agreement between cardiac implant RDI and the AHI assessed by respiratory polygraphy without sleep assessment. They found a 22% prevalence of severe SA. The European DREAM study^2^ evaluated a population of 40 patients newly implanted for symptomatic sinus node dysfunction, permanent atrio-ventricular block, or severe heart failure by using another cardiac implant brand algorithm. In this study, the prevalence of moderate to severe SA was up to 59%, with 27% of the patients in the severe spectrum of the disease. Take together; these studies demonstrate that the strategy of SA diagnosis using cardiac implants is feasible and reliable in different cardiac disease populations. One of the strengths of our study is to separate central and obstructive apneas/hypopnea and to use recommended definitions for hypopneas which was not the case in previous studies.

Regarding the impact on outcome of SA diagnosis, Mazza *et al*.^12^ have recently investigated SA using the Apneascan algorithm in 160 patients implanted for symptomatic bradycardia. In this population, SA was independently associated with a higher risk of AF (AF Burden ≥ 6 h/day) and new-onset AF. In addition, the existence of severe SA allowed to identify patients who were 2-fold more likely to experience an AF episode in the next 3 months. The clinical impact of SDB on the incidence of appropriate ICD therapy in patients with heart failure and reduced ejection fraction has recently been explored in a meta-analysis^11^. The results show an increased risk of appropriate ICD therapy among patients with SDB compared with those without. The results of this analysis support the emerging evidence concerning the adverse impact of SDB on HF outcomes by triggering severe arrhythmias. A close monitoring of SA in these patients by ApneaScan might help to improve arrhythmias management and in turn improve prognosis.

**Figure 5:**
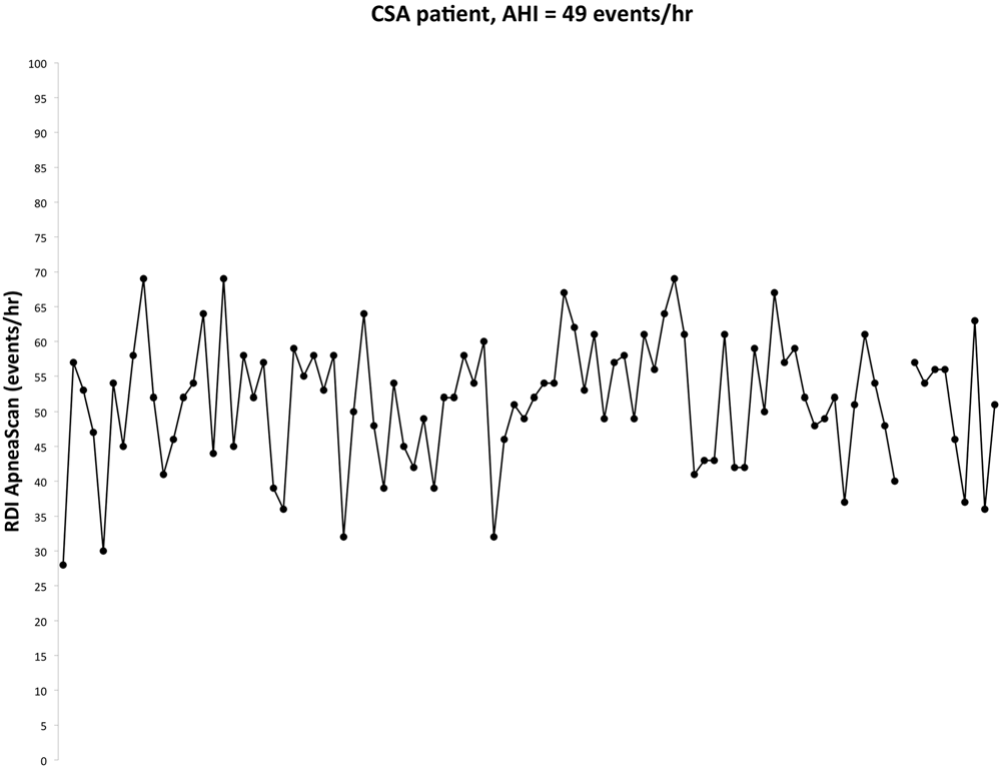
Night to night variability in AHI in a patient with CSA over the 3 month study period.

Moubarak *et al*.^13^ analyzed 58 patients implanted with pacemakers equipped with a transthoracic impedance sensor for measuring RDI and found that RDI could be measured on 98% of nights. Mean RDI was 19.9 ± 12.7 and most patients (90%) had at least 1 night with RDI > 20. This study shows that RDI is subject to a high degree of variability as shown by the mean individual patient night-to-night RDI variability (19% ± 21%). This is again a relevant point favoring such a night after night monitoring versus an in-lab unique hospital test made just on one single night. This night to night and intra night variability is especially prevalent in cardiac failure patients with central sleep apnea^14^. The device used in the present study allowed tracking patients RDI night-to-night over the 3- month study period. Figure 5 illustrates a typical recording of a patient with CSA and an AHI-PSG of 49 events/hr with a RDI on ApneaScan fluctuating from 28–69 events/hr. This type of follow-up has the potential to identify exacerbations in chronic cardiac failure patients as acute cardiac failure is nearly systematically associated with an increase in CSA burden^15^.

Our findings have several clinical implications. First of all, patients with an ICD or CRT-D device equipped with such an algorithm can be continuously and automatically monitored during their sleeping time. Second, the use of this automatic feature does not require any specific work to be planned and done in the hospital for the diagnosis, thus positively impacting the use of the available resources. Pulse oximetry data can also be used to identify sleep apnea^16^. However, the undeniable benefit of using the pacemaker is that no additional probe or sensor is needed, patients do not require an additional intervention and there are no waiting lists to be screened for sleep apnea. Furthermore, the device can periodically transmit the data about SA, cardiac arrhythmias, and many other parameters and trends, so that paramedical staff performing remote follow-up of the implanted patients can review the data and share with the cardiologist in case a clinical action is required^17^. Sleep apnea diagnosis and follow-up should certainly be included in the overall telemonitoring strategy of patients with cardiac implants.

### Study limitations

Due to relatively limited patient population enrolled in this study we could not perform a clinically meaningful analysis of the SA trend versus the arrhythmic events (atrial fibrillation and appropriate therapies from the ICD, Ventricular Tachycardia, Ventricular Fibrillation, and inappropriate therapies) stored by the devices during the 3 month follow-up period. Furthermore, the number of patients included in the present study did not allow us to achieve a high specificity. However, for a screening method, sensitivity is the key issue to avoid false negatives and the results we obtained with the current sample size yielded robust results. We also were unable to carry out subgroup analyses based on the type of sleep apnea (OSA versus CSA) and based on sex. Additional prospective and large scale studies are needed to clarify these relationships.

## Conclusions

The AIRLESS study shows that a transthoracic impedance sensor (ApneaScan algorithm) implemented in the ICDs for SA detection through the assessment of transthoracic impedance is a reliable tool to identify patients suffering of severe sleep disorders. The availability of this new technology might improve the clinical management of this patient population. Larger and long-term prospective studies are needed to collect data on the clinical impact.
